# Catalyst-Assisted Large-Area Growth of Single-Crystal *β*-Ga_2_O_3_ Nanowires on Sapphire Substrates by Metal–Organic Chemical Vapor Deposition

**DOI:** 10.3390/nano10061031

**Published:** 2020-05-28

**Authors:** Chunyang Jia, Dae-Woo Jeon, Jianlong Xu, Xiaoyan Yi, Ji-Hyeon Park, Yiyun Zhang

**Affiliations:** 1 R&D Center for Solid-state Lighting, Institute of Semiconductors, Chinese Academy of Sciences, Beijing 100083, China; jiachunyang16@mails.ucas.edu.cn (C.J.); spring@semi.ac.cn (X.Y.); 2 Center of Materials Science and Optoelectronics Engineering, University of Chinese Academy of Sciences, Beijing 100049, China; 3 Korea Institute of Ceramic Engineering and Technology, 15-5, Chungmugong-dong, Jinju-si, Gyeongsongnam-do 52851, Korea; dwjeon@kicet.re.kr; 4 Jiangsu Key Laboratory for Carbon-Based Functional Materials & Devices, Institute of Functional Nano & Soft Materials (FUNSOM), Soochow University, Suzhou 215123, China; xujianlong@suda.edu.cn

**Keywords:** *β*-Ga_2_O_3_, nanowires, MOCVD

## Abstract

In this work, we have achieved synthesizing large-area high-density *β*-Ga_2_O_3_ nanowires on c-plane sapphire substrate by metal–organic chemical vapor deposition assisted with Au nanocrystal seeds as catalysts. These nanowires exhibit one-dimensional structures with Au nanoparticles on the top of the nanowires with lengths exceeding 6 μm and diameters ranging from ~50 to ~200 nm. The *β-*Ga_2_O_3_ nanowires consist of a single-crystal monoclinic structure, which exhibits strong ($\bar{2}$01) orientation, confirmed by transmission electronic microscopy and X-ray diffraction analysis. The PL spectrum obtained from these *β*-Ga_2_O_3_ nanowires exhibits strong emissions centered at ~360 and ~410 nm, respectively. The energy band gap of the *β*-Ga_2_O_3_ nanowires is estimated to be ~4.7 eV based on an optical transmission test. A possible mechanism for the growth of *β*-Ga_2_O_3_ nanowires is also presented.

## 1. Introduction

*β*-gallium oxide (*β*-Ga_2_O_3_) is emerging as an important ultrawide bandgap semiconductor for widespread applications such as gas sensors [1,2], power electronics [3,4] and solar-blind photodetectors, due to its wider energy band gap (E_gv_ = ~4.8 eV) and higher breakdown field strength (~8 MV/cm) [5,6,7] when compared to its counterparts SiC and GaN. As great effort has been made to achieve high-quality thin-film *β*-Ga_2_O_3_ on homo/hetero substrates, the controllable growth of one-dimensional *β*-Ga_2_O_3_ nanowires is promising and urgent, as it would open up new possibilities and opportunities in the scaling down of devices to achieve ultracompact nanoscale electronic devices. Moreover, compared to other phases of Ga_2_O_3_, such as *α*-, *γ*-, *δ*- and *κ*-phase, *β*-Ga_2_O_3_ is the most stable and robust structure_._ This feature suggests *β*-Ga_2_O_3_-based nanoscale devices can function stably when used in extreme environments [8]. To address this, a variety of techniques have been employed previously to synthesize *β*-Ga_2_O_3_ nanowires, including physical evaporation [9,10,11,12], arc-discharge [13], vapor–liquid–solid method (VLS) [14,15,16], microwave plasma [17], chemical vapor deposition (CVD) [18,19,20], metalorganic chemical vapor deposition (MOCVD) [21,22,23], and thermal reduction method [24,25], which in all exhibit various limitations in the terms of either growth speed, large-area epitaxy or material quality. More importantly, in practice, processing of nanowire devices usually calls for precisely controlled growth of a highly-oriented nanowire array with a good uniformity in the size of individual nanowires. In the present work, a new concept of growing large-area single-crystal *β*-Ga_2_O_3_ nanowires on sapphire substrate was demonstrated by using predeposited Au nano seeds as catalysts via MOCVD, which may potentially shed some light on solving this problem. We also studied the growth process of *β*-Ga_2_O_3_ nanowires by performing multiple characterizations to observe the morphology, crystal structure, crystalline quality, and the growth direction of *β*-Ga_2_O_3_ nanowires by scanning electron microscopy (SEM), transmission electron microscopy (TEM), selected area electron diffraction(SAED), X-ray diffraction (XRD), Raman spectroscopy, and photoluminescence (PL) spectroscopy.

## 2. Materials and Methods

### 2.1. Growth of β-Ga_2_O_3_ Nanowires

**Figure 1:**
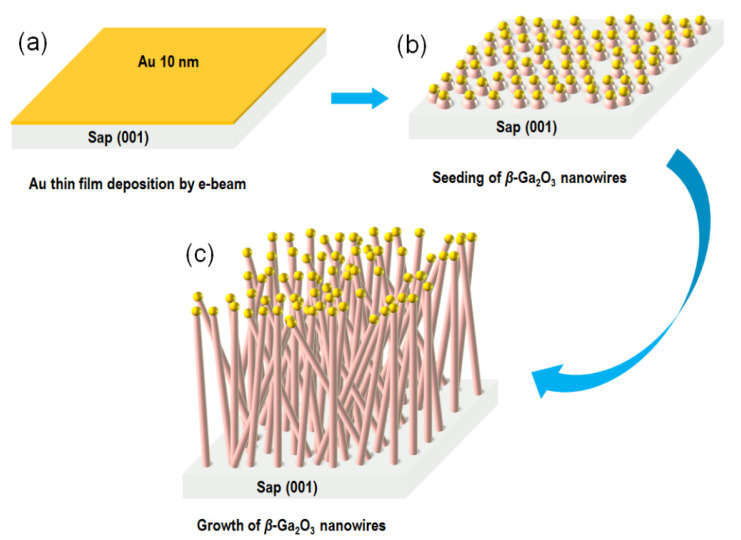
Schematic diagram depicting growth process of single-crystal *β*-Ga_2_O_3_ nanowires on *c*-plane sapphire substrates by metalorganic chemical vapor deposition (MOCVD). (**a**) Au thin-film deposition. (**b**) Seeding of *β*-Ga_2_O_3_ nanowires. (**c**) Growth of *β*-Ga_2_O_3_ nanowires.

Before the Au evaporation, the sapphire substrate was cleaned in an ultrasonic bath to remove the contaminations for 5 min with organic solvents including acetone and methanol, respectively. Then, the substrate was dried using N_2_ gun and loaded to the MOCVD reactor. A commercial horizontal-flow MOCVD system was used to grow *β*-Ga_2_O_3_ nanowires on *c*-plane sapphire substrates. A conventional trimethylgallium (TMGa) bubbler and high-purity deionized (DI) vapor were used as the gallium and oxygen precursors, respectively. H_2_ was used as the carrier gas. To start the growth, 10 nm thick Au thin film was first deposited on the sapphire substrate by e-beam evaporation. Then the wafer was loaded in the MOCVD reactor. TMGa was flowed for 1 min to predeposit gallium on the substrate before introducing the water vapor. *β*-Ga_2_O_3_ nanowires were synthesized at a temperature of 690 °C with VI/III flow ratio of 300. The schematic diagram depicting catalyst-assisted large-area growth of single-crystal *β-*Ga_2_O_3_ nanowires on sapphire substrate by MOCVD is illustrated in Figure 1.

**Figure 2:**
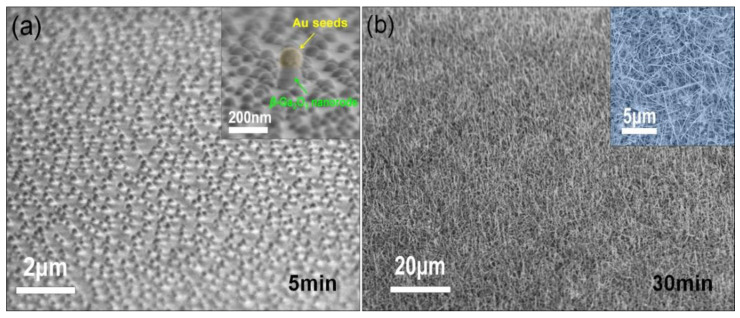
Scanning electron microscopy (SEM) images illustrating the growth process of *β*-Ga_2_O_3_ nanowires (**a**) self-assembled Au nanocrystals as catalysts while sitting on *β*-Ga_2_O_3_ nanorods after 5 min growth; inset shows a close-up SEM image of a *β*-Ga_2_O_3_ nanorod under an Au nanocrystal after 5 min growth. (**b**) 45 degree tilted-view SEM image of *β*-Ga_2_O_3_ monoclinic nanowires after 30 min growth; inset is the bird-view SEM image of *β*-Ga_2_O_3_ nanowires after 30 min growth.

Before the root of *β*-Ga_2_O_3_ nanowires was grown, due to the surface tension effect, predeposited Au thin film self-assembled into nano islands that were randomly distributed all over the sapphire substrate as the reactor chamber temperature got higher, which could be used as catalysts in the growth of *β*-Ga_2_O_3_ nanowires on sapphire, with Au remaining in liquid form at this stage. [14,26] This could be apparently confirmed by the SEM images showing the growth process of *β*-Ga_2_O_3_ nanowires after 5 min and 30 min, as shown in Figure 2. The inset of Figure 2a clearly illustrates a *β*-Ga_2_O_3_ nanorod underneath an Au nanocrystal after 5 min growth. This growth process lasts as the nanowires get longer, while their diameters are highly consistent with the sizes of the Au nanocrystals, by which the growth direction selectivity and aspect ratio are greatly improved.

### 2.2. Characterization Methods

The growth process was monitored by taking the samples out of the MOCVD reactor to check the evolution of *β*-Ga_2_O_3_ nanowires with SEM. As the growth finished, the orientation of *β*-Ga_2_O_3_ nanowires were investigated by SEM (Hitachi S-4800), TEM (FEI Tecnai-G2-F20), Raman spectroscopy (HORIBA Scientific) and X-ray diffraction (Bede X-ray Metrology, 40 kV, λ: ~1.54 Å). A 532 nm green laser was used as the excitation source for the Raman test through an optical setup with an objective of 50×. In each scan, the integration time was set to be 5 min for collecting Raman signals from *β*-Ga_2_O_3_ nanowires. The energy band gap of *β*-Ga_2_O_3_ nanowires was estimated after a numerical fitting based on the transmittance spectrum using wideband light sources (200~800 nm) as the incidence. The transitions of excited carriers were analyzed to investigate the defects and their impact on the emission characteristics for *β*-Ga_2_O_3_ nanowires based on room temperature PL spectra. A 248 nm excimer laser was used as an optical pumping source and PL signals were collected by a prefocus lens and coupled into the entrance slit of a 500 mm spectrograph, dispersed by a 1200 L/mm grating to a cooled charged-coupled device (CCD), offering an optical resolution about 0.1 nm. As a comparison, a native *β*-Ga_2_O_3_ substrate was also included in the PL test.

## 3. Results and Discussion

**Figure 3:**
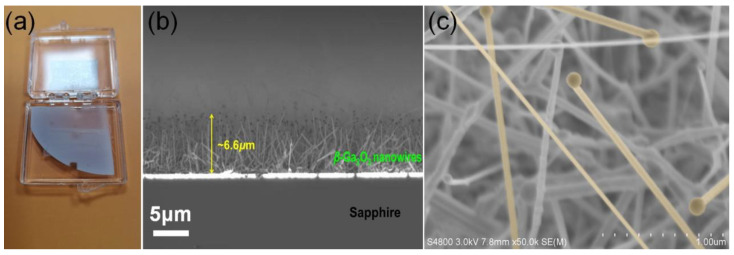
(**a**) A quarter of 2-inch as-grown *β*-Ga_2_O_3_ nanowires on sapphire wafer. (**b**) Cross-sectional SEM image of the as-grown *β*-Ga_2_O_3_ nanowires. (**c**) High-magnification SEM image of nanowires.

An optical image in Figure 3a presents a quarter of a 2-inch sapphire wafer with *β*-Ga_2_O_3_ nanowires synthesized by MOCVD. Gray color of the wafer indicates that the nanowires are uniformly distributed on the sapphire substrate over a large area. These nanowires exhibit a nominal height of ~6.6 μm, as shown in the cross-sectional SEM image of *β*-Ga_2_O_3_ nanowires on sapphire in Figure 3b. A higher magnification SEM image in Figure 3c reveals the fairly vertically-oriented growth direction of *β*-Ga_2_O_3_ nanowires, with diameters ranging from ~50 nm to ~200 nm, indicating that the straight and long nanowires exhibit a very large aspect ratio. This is particularly true at the beginning of growth, as shown in the Figure 2b. In fact, the diameters of *β*-Ga_2_O_3_ nanowires are highly depended on the sizes of Au seeds before the growth, which suggests the feasibility to tune the size and density for as-grown *β*-Ga_2_O_3_ nanowires by depositing a thinner Au film for smaller and sparser Au nanoseeds. In addition, we can clearly observe that the spherical-shaped Au nanoparticles are sitting on the stem of the nanowire at the tips, which again confirms the growth mechanism as described before. The growth rate of nanowire in the vertical direction can be roughly estimated as 200 nm/min.

**Figure 4:**
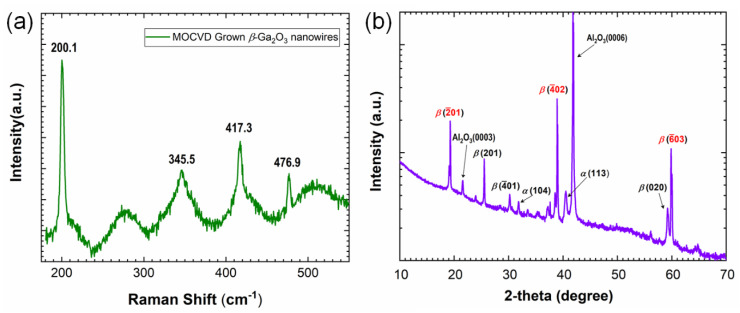
(**a**) Raman and (**b**) X-ray diffraction (XRD) spectra of *β*-Ga_2_O_3_ nanowires.

Raman analysis was performed to analyze the crystal structure on typical *β*-Ga_2_O_3_ nanowire samples. It can be seen that strong and sharp Raman peaks from crystalline Ga\O bonds are centered at 200.1, 345.5, 476.9 and 417.3 cm^−1^ in Figure 4a. This spectrum demonstrates that the nanowires present the monoclinic *β*-Ga_2_O_3_ phase and have a very good crystal quality [27,28]. Meanwhile, XRD was used to examine the crystal structure of the large-area *β-*Ga_2_O_3_ nanowires grown by MOCVD. A typical XRD pattern of as-grown sample is shown in Figure 4b. The peaks at 21° and 41° correspond to the {0001} diffraction peaks of the sapphire substrate. In addition, three dominant diffraction peaks at 18.9°, 38.4° and 59.2° are observed, which correspond to the ($\bar{2}$01), ($\bar{4}$02), and ($\bar{6}$03) planes of *β-*Ga_2_O_3_, respectively. It illustrates that the *β-*Ga_2_O_3_ nanowires are mostly ($\bar{2}$01)-oriented, which is almost paralleled to the sapphire (0001) plane [29]. Three much weaker peaks correspond to the (201), ($\bar{4}$01), and (020) planes of *β-*Ga_2_O_3_. It again affirms that the as-grown vertically-oriented *β-*Ga_2_O_3_ nanowires have the monoclinic structure (*β-*Ga_2_O_3_ (a = 12.12–12.34, b = 3.03–3.04, c = 5.78–5.87)), according to the Joint Committee on Powder Diffraction Standards (JCPDS) powder diffraction file No. 76-0573. [29,30]. The growth temperature (690 °C) may contribute to the diffraction peaks of the *α*-phase Ga_2_O_3_ nanowires in Figure 4b [31].

**Figure 5:**
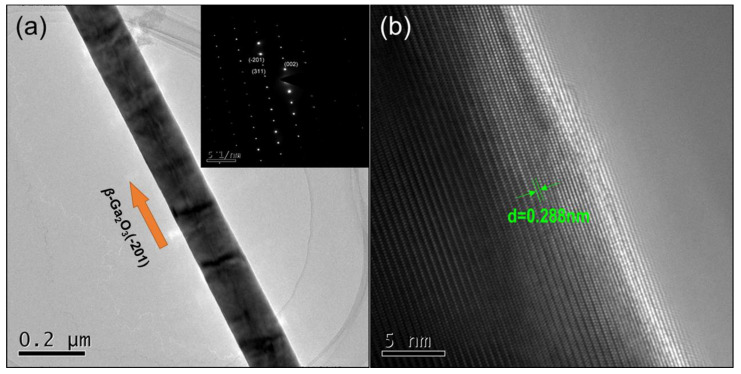
(**a**) Transmission electron microscopy (TEM) image of an individual *β*-Ga_2_O_3_ nanowire. The typical electron diffraction pattern is in the inset. (**b**) High-resolution TEM image of the *β*-Ga_2_O_3_ nanowires with uniform lattice fringes.

In order to further investigate the detailed crystal structure of the as-grown sample, Figure 5a presents a typical TEM image of an individual synthesized *β-*Ga_2_O_3_ nanowire with a diameter of about 80 nm. The selected area electron diffraction (SAED) shown in the inset of Figure 5a confirms that the synthesized sample shows perfect crystallinity of monoclinic *β*-Ga_2_O_3_. Figure 5b presents a high resolution TEM (HRTEM) image of the *β*-Ga_2_O_3_ nanowires. The lattice fringes with a *d* spacing of 0.288 nm are observed, which corresponds to the (004) lattice planes of *β*-Ga_2_O_3_. The *β*-Ga_2_O_3_ nanowires of the same direction as the white arrow grow along the ($\bar{2}$01) direction, which is consistent with the results given in Figure 5a.

**Figure 6:**
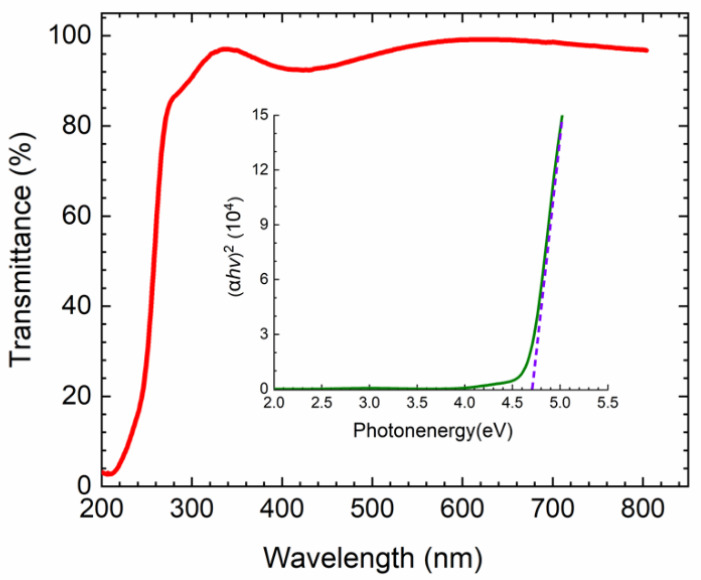
Optical transmission spectrum of the *β*-Ga_2_O_3_ nanowires. (*αhυ*)^2^ versus photon energy plots of the *β*-Ga_2_O_3_ nanowires in the inset.

Figure 6 presents the optical transmittance of the *β*-Ga_2_O_3_ nanowires. It can be clearly observed that the band-edge absorption for the as-grown *β*-Ga_2_O_3_ nanowires is at about 275 nm, from which the band gap can be roughly estimated as 4.53 eV by using the equation: *E*_g_ = 1240/*λ*. Moreover, it has a much lower absorption in the visible region when compared with the sharp band-edge absorption in the deep ultraviolet region below 280 nm, suggesting great potential for being used as solar-blind photodetectors for these nanowires. For a more accurate estimate, the optical band gap of the *β*-Ga_2_O_3_ nanowires is calculated by extrapolating the linear portion of the square of absorption coefficient against photon energy using the equation:

(1) $${\left(\alpha hv\right)}^{2}=B\cdot \left(hv-E_{g}\right)$$

where *α* is absorption coefficient, *h* is the Planck’s constant, and *υ* is the frequency of the incident light, and B is a constant [7,32,33,34]. The band gap of *β*-Ga_2_O_3_ nanowires is estimated to be ~4.7 eV as shown in the inset of Figure 6. This value is in agreement with the band gaps from experiments ranging from 4.6 to 4.9 eV as reported by others [29]. It is also consistent with theoretical predictions of a direct band gap at Γ-point (Eg(Γ-Γ), ~4.8 ± 0.1 eV) and indirect band gap at L-point (Eg(Γ-L), ~4.7 ± 0.1 eV) by quasiparticle self-consistent GW calculations for *β*-Ga_2_O_3_ [35].

**Figure 7:**
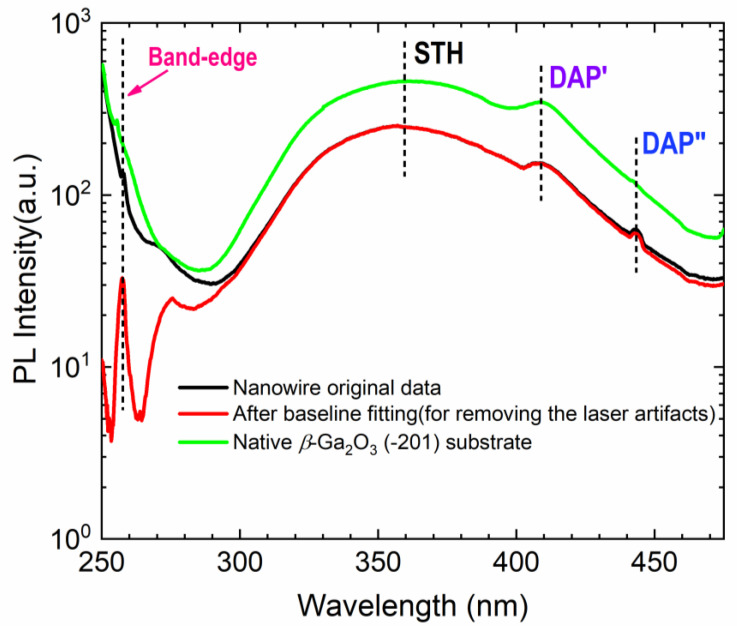
Room temperature photoluminescence (PL) spectra of *β*-Ga_2_O_3_ nanowires and native *β*-Ga_2_O_3_ substrate at the same optical excitation conditions.

Figure 7 shows the PL spectrum obtained from *β*-Ga_2_O_3_ nanowires and native *β*-Ga_2_O_3_ (-201) substrate at room temperature at the same pumping conditions. For removing the artifacts from the laser sources, the PL spectrum from the nanowires is baseline-fitted. In the baseline fitting, to identify the intrinsic emission, the PL spectrum of nanowires is separated from the laser excitation background, as the intrinsic emissions of *β*-Ga_2_O_3_ is greatly overlapped with laser excitation background (see Figure 7). Emission spectral peaks centered at ~257 nm and ~275 nm can be observed, which correspond with the band-edge transitions of excited carriers that originate from the anisotropy of the monoclinic phase [36]. Aside from the band-edge emission, *β*-Ga_2_O_3_ nanowires exhibit two distinct spectral peaks at ~360 nm and ~410 nm, respectively, which are consistent with the PL spectral peaks of native *β*-Ga_2_O_3_ (-201) substrate. The peak at 360 nm is originated from recombination processes caused by self-trapped holes (STH), which is an intrinsic feature and is not due to defects. The spectral peak located at 410 nm is related to the recombination of donor–acceptor pairs (DAP), which is a result of electrons tunneling from the donor to the acceptor when photo-generated carriers are captured by donors or acceptors in *β*-Ga_2_O_3_. A weak spectral peak can be identified at ~440 nm for the *β*-Ga_2_O_3_ nanowires, corresponding to the blue band emission that strongly correlates with oxygen vacancy concentration [37]. Overall, the PL spectrum from *β*-Ga_2_O_3_ nanowires is highly consistent with that from native *β*-Ga_2_O_3_ substrate, which suggests high material quality of as-grown *β*-Ga_2_O_3_ nanowires.

## 4. Conclusions

In summary, we have presented a new method to grow large-area single-crystal monoclinic *β*-Ga_2_O_3_ nanowires by MOCVD. Assisted by Au nanocrystal seeds as catalysts, the growth of nanowires exhibited a very good selectivity in the ($\bar{2}$01) direction, which demonstrates an array of >6 μm long one-dimensional nanowires. Diameters of the nanowires are highly depended on the sizes of Au nano particles, ranging from ~50 to ~200 nm. The room temperature PL spectrum obtained from *β*-Ga_2_O_3_ nanowires exhibits strong emissions centered at ~360 and ~410 nm, representing the recombination processes via electrons/self-trapped holes and donor–acceptor pairs, respectively, while the blue band emission was greatly suppressed with less O vacancies in *β*-Ga_2_O_3_ nanowires. The energy band gap of the *β*-Ga_2_O_3_ nanowires is estimated to be 4.7 eV according to the optical transmission test. Our method suggests a great potential in realizing controllable growth of *β*-Ga_2_O_3_ nanowires by predepositing highly-ordered Au nanoplates for arrayed growth. This work provides a new thought on the synthesis of high-quality *β*-Ga_2_O_3_ nanowires that facilitates the scaling down in the perspective of fabricating *β*-Ga_2_O_3_-based field-effect transistors and solar-blind photodetectors.
